# An automatic system for recognizing fly courtship patterns via an image processing method

**DOI:** 10.1186/s12993-024-00231-4

**Published:** 2024-03-16

**Authors:** Ching-Hsin Chen, Yu-Chiao Lin, Sheng-Hao Wang, Tsung-Han Kuo, Hung-Yin Tsai

**Affiliations:** 1 Department of Power Mechanical Engineering, National Tsing Hua University, Hsinchu, 30013 Taiwan; 2 Department of Life Science, National Tsing Hua University, Hsinchu, 30013 Taiwan; 3 Institute of Systems Neuroscience, National Tsing Hua University, Hsinchu, 30013 Taiwan; 4 Brain Research Center, National Tsing Hua University, Hsinchu, 30013 Taiwan

**Keywords:** Aging, Complex behavior, Behavior monitoring, Image processing, Fly courtship

## Abstract

Fruit fly courtship behaviors composed of a series of actions have always been an important model for behavioral research. While most related studies have focused only on total courtship behaviors, specific courtship elements have often been underestimated. Identifying these courtship element details is extremely labor intensive and would largely benefit from an automatic recognition system. To address this issue, in this study, we established a vision-based fly courtship behavior recognition system. The system based on the proposed image processing methods can precisely distinguish body parts such as the head, thorax, and abdomen and automatically recognize specific courtship elements, including orientation, singing, attempted copulation, copulation and tapping, which was not detectable in previous studies. This system, which has high identity tracking accuracy (99.99%) and high behavioral element recognition rates (> 97.35%), can ensure correct identification even when flies completely overlap. Using this newly developed system, we investigated the total courtship time, and proportion, and transition of courtship elements in flies across different ages and found that male flies adjusted their courtship strategy in response to their physical condition. We also identified differences in courtship patterns between males with and without successful copulation. Our study therefore demonstrated how image processing methods can be applied to automatically recognize complex animal behaviors. The newly developed system will largely help us investigate the details of fly courtship in future research.

**Supplementary Information:**

The online version contains supplementary material available at 10.1186/s12993-024-00231-4.

## Introduction

Courtship is a common behavior that occurs in several animal species. An individual, usually a male, displays a series of actions to attract a mate for copulation. Because of its direct impact on mating success, sexual selection strongly affects traits related to courtship and results in a variety of behavioral manifestations among different species [13]. Courtship signals occur in many sensory modalities. According to the handicap hypothesis [29], honest indicators should be costly to maintain or risky for predation. Therefore, performing courtship correctly and at an appropriate time is a critical decision for animals not only in reproduction but also in survival.

In fruit flies, males display courtship steps (or behavioral elements) before successful copulation, including orientation, tapping, licking, singing, and attempted copulation [7]. During this courtship ritual, several sexual signals are exchanged between the two sexes. Females evaluate male performance and eventually decide to mate or leave the male. As one of the standard genetic model organisms, fly courtship behavior has been an ideal model for addressing proximate or ultimate questions in different biological fields. For example, multiple genes involved have been identified [7]. The neural circuits from pheromone perception to motor output of wing vibration have been mapped [5, 28]. The roles of courtship in speciation have also been addressed in several evolutionary studies [8, 25]. However, even though overall courtship has been investigated extensively, the difference or importance of each behavioral element has mostly been ignored in previous studies. Unfortunately, examining the details of these behavioral elements would require enormous video watching and analyses.

Several programs have been designed to automatically detect locomotion [9, 18, 19, 22, 26] or identify specific behaviors, such as flight or even social interaction, in flies [11, 12]. One of the main challenges for monitoring multiple flies simultaneously is to distinguish flies’ identities when they are on top of each other, especially in overlapping situations. The identity may be swapped after overlapping, and this incorrect tracking result will continuously exist in the remainder of the tracking process. Many studies have integrated clustering methods to address this problem [3, 6, 15]. However, these methods can only work reliably in a partially overlapping situation. To approach the completely overlapping issue, identifying and matching body parts (head, thorax, and abdomen) can be a solution for assigning fly identities and minimizing the wrong identification caused by overlapping situations.

The core monitoring techniques can be generally divided into two methods: machine learning [12] and image processing [1, 18, 19, 22]. Machine learning methods with no requirement for behavioral definition are often used to identify complex behaviors. For example, based on the locomotion information generated by Ctrax [1], an open-source tool for detecting fly locomotion, JAABA [12] can apply the machine learning method (boosting algorithm) to train behavior classifiers, and Jiang et al. [11] establish a deep neural network system for recognizing complex social behaviors. On the other hand, developing a model in machine learning requires a great amount of manually labeled training data, which is often time-consuming and laborious. In addition, the requirement for graphics cards is usually high and not friendly to ordinary researchers if the model is complex and involves a large dataset. More critically, machine learning relies heavily on previous training materials. When new environments are introduced for a variety of reasons, such as new cameras, light sources, or new arena designs, the model will need to be retrained with a large amount of manually labeled data, which will once again consume considerable time and effort. In contrast to the disadvantages of machine learning methods, the hardware requirement for image processing methods is much lower. When the environment changes, only the values of the behavioral definitions need to be altered for new data analyses. Therefore, this method provides an alternative approach to establish an easier, cheaper and potentially more robust behavioral recognition system.

In this study, to comprehensively examine fly courtship behaviors, we applied the image processing technique to develop an automatic system to recognize and analyze male courtship behavioral elements, including copulation, attempted copulation, singing, orientation and tapping, which was not detectable by previous programs. Additionally, as there has been no specific investigation into identity tracking errors caused by overlapping features between male and female flies, our study proposed a method that contrasts the torso region of flies to effectively reduce identity recognition errors during tracking. The analyzed data provided information on total courtship, the proportion of courtship elements, and the transition of courtship elements. By taking advantage of this system, we easily examined multiple courtship events and detected changes in courtship behaviors across ages. We also compared the behaviors of males with successful copulation and males who failed to mate. In summary, our developed system allowed us to investigate the details of courtship elements, which could be important signals for female mating decisions in fruit flies. More importantly, this project demonstrated that, with appropriate definitions, image processing procedures can be used to establish an easy and applicable system for recognizing complex behaviors.

## Methods and materials

### Experimental setup

**Fig. 1:**
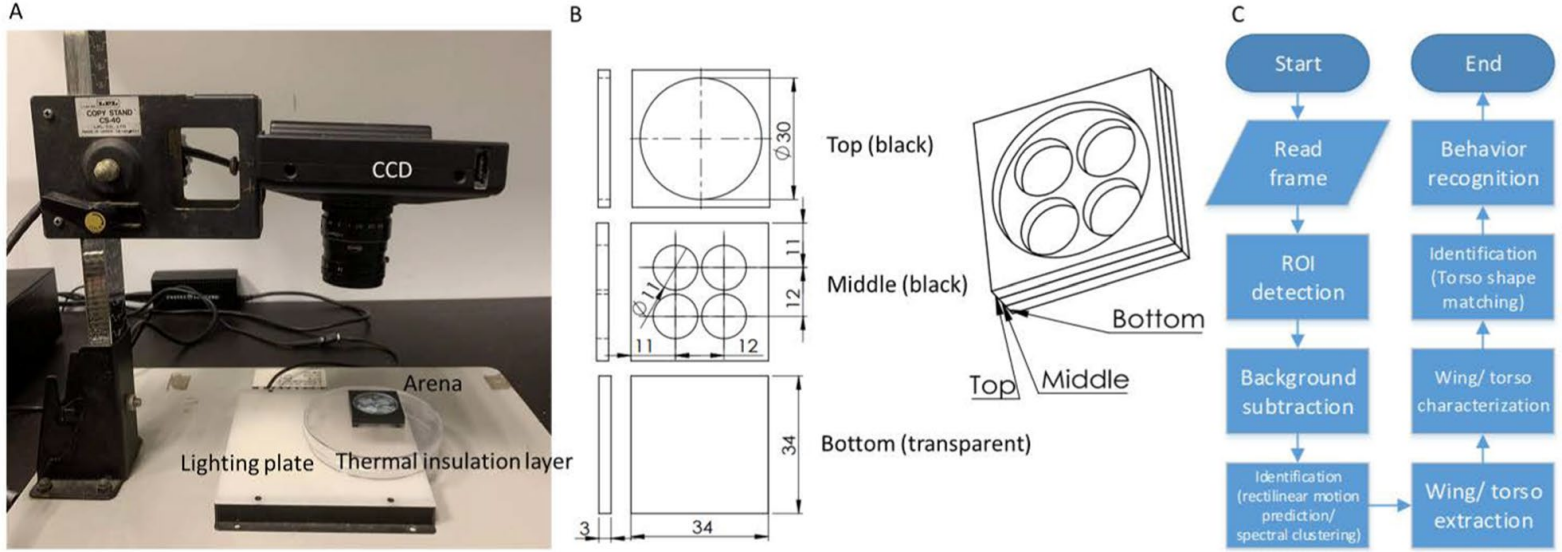
Equipment setup, arena design, and analysis procedure. **A** Experimental setup. The CCD captures frames above the arena. A glass plate was set between the arena and the lighting plate as a thermal insulation layer. **B** In the arena design, the fly can move in a cylindrical space of 11 mm in diameter and 3 mm in height. All four tests can be performed simultaneously. **C** An overview of the flow chart of the software

The experimental setup consisted of a camera (IL5 high-speed camera, Fastec), a lens (LM25HC, Kowa), an arena, a light plate, and a thermal insulation layer (Fig. 1A). The camera above the arena captured images at 1100 × 1100 pixels at 24 frames/second. The light plate was set under the arena (Fig. 1B). A backlighting design was applied to separate objects from the background smoothly. Since a long recording duration would cause a temperature increase in the environment, a thermal insulation layer was used to isolate the heat from the light plate.

### Software overview

A series of steps were proposed in this study to characterize the flies (Fig. 1C). It is important to note that when the details of the experimental parameter settings for this study are described in the Supplementary Information (Additional file 1), users have the flexibility to adjust these values in the program settings based on their specific circumstances.

1. **Frame reading:** The frame was loaded into the system first.
2. **Region of interest (ROI) detection:** There were 4 regions in one frame. The Hough transform was applied to detect these four circles in the frame.
3. **Background subtraction:** This step yielded the silhouettes of flies in each arena.
4. **Identification:** While two flies overlapped, which can be detected by the number of silhouettes, spectral clustering was applied to separate the overlapping regions. The separated regions can then be assigned to the corresponding identities by rectilinear motion prediction.
5. **Wing/torso extraction:** Next, the wing and torso were extracted from each silhouette and characterized.
6. **Wing/torso characterization:** The position of the fly, the heading direction, and the wing extension angles can be determined in this step.
7. **Identity checkup:** During overlapping events, the identities were assigned by rectilinear motion prediction in the (4) identification step. After the two flies were separated, torso shape matching was executed to verify the identity of the flies. The torso was segmented into head, thorax, and abdomen parts and can be used to assign the identities of flies directly.
8. **Behavior recognition:** The final step was to apply defined parameters to identify behavioral elements.

### Background subtraction

**Fig. 2:**
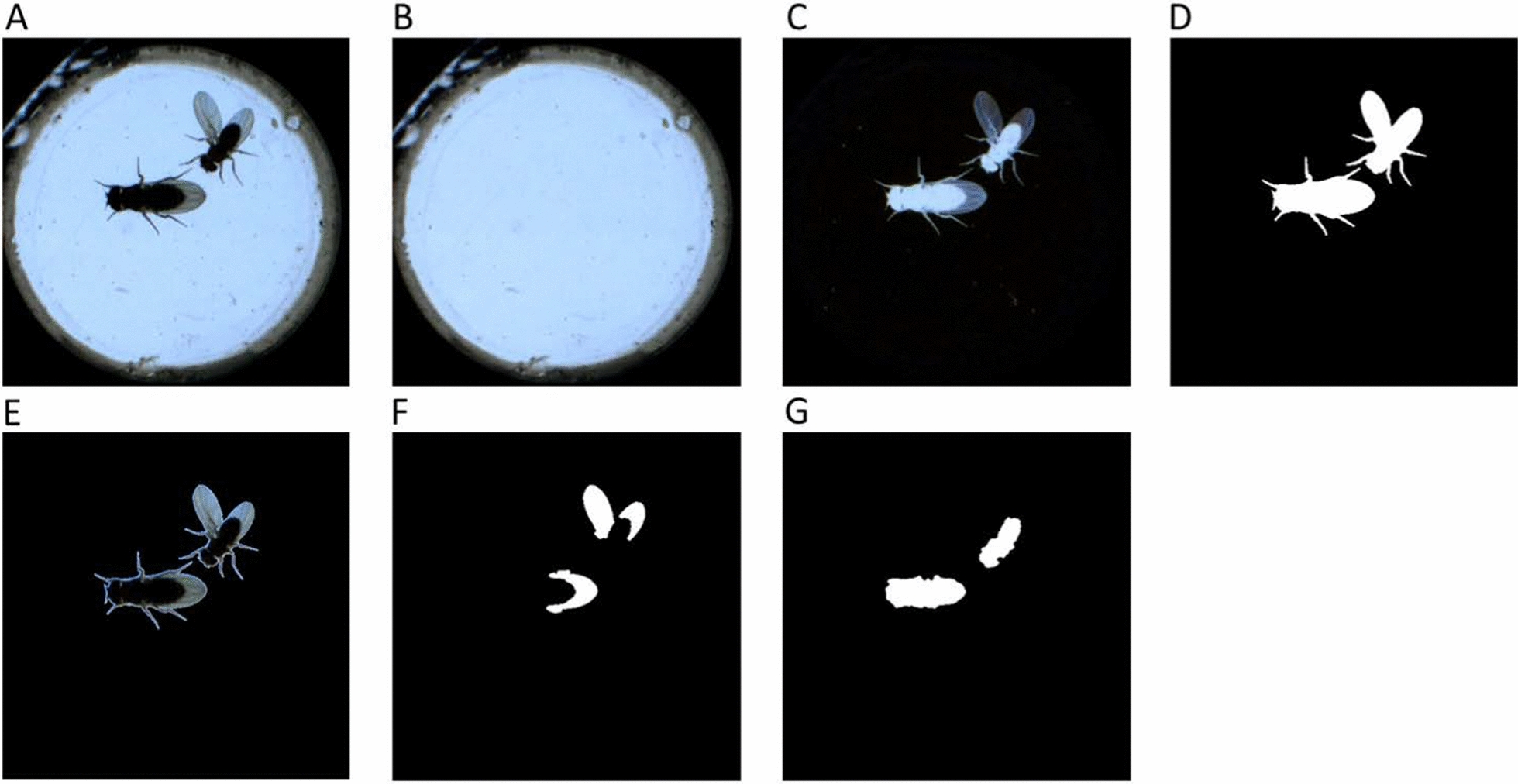
The demonstration of each step in wing/torso detection. **A** Raw image. **B** Background generated by a spatial maximum filter. **C** Background subtraction results from (**B**) and (**C**). **D** Binary image of (**C**). **E** Foreground image of (**D**). **F** Wing extraction result. **G** Torso extraction result

The background subtraction method was applied to obtain the initial silhouette of the flies. Figure 2A shows the raw data of a frame. A clear background was generated by a spatial maximum filter [4] (Fig. 2B). As the subtraction process was applied (Fig. 2C), a clear silhouette could be collected by thresholding (Fig. 2D).

### Wing/torso extraction

The foreground image (Fig. 2E) was obtained by multiplying the silhouette (Fig. 2D) and the raw image (Fig. 2A). Wings can be extracted by applying the following equation with morphological processing (Fig. 2F):

$$Region\,of\,wings=round\left[\frac{Fig. 2E(R\,space)}{80}\times \frac{Fig. 2E(G\,space)}{80}\times \frac{Fig. 2E(B\,space)}{80}\right]>0$$

R, G, and B in the equation denote the pixel intensities in the red, green, and blue color spaces, respectively. The torso is then obtained by subtracting the region of the wings from the silhouette via morphological processing (Fig. 2G).

### Wing/torso characterization - Fly’s position and heading (torso processing)

**Fig. 3:**
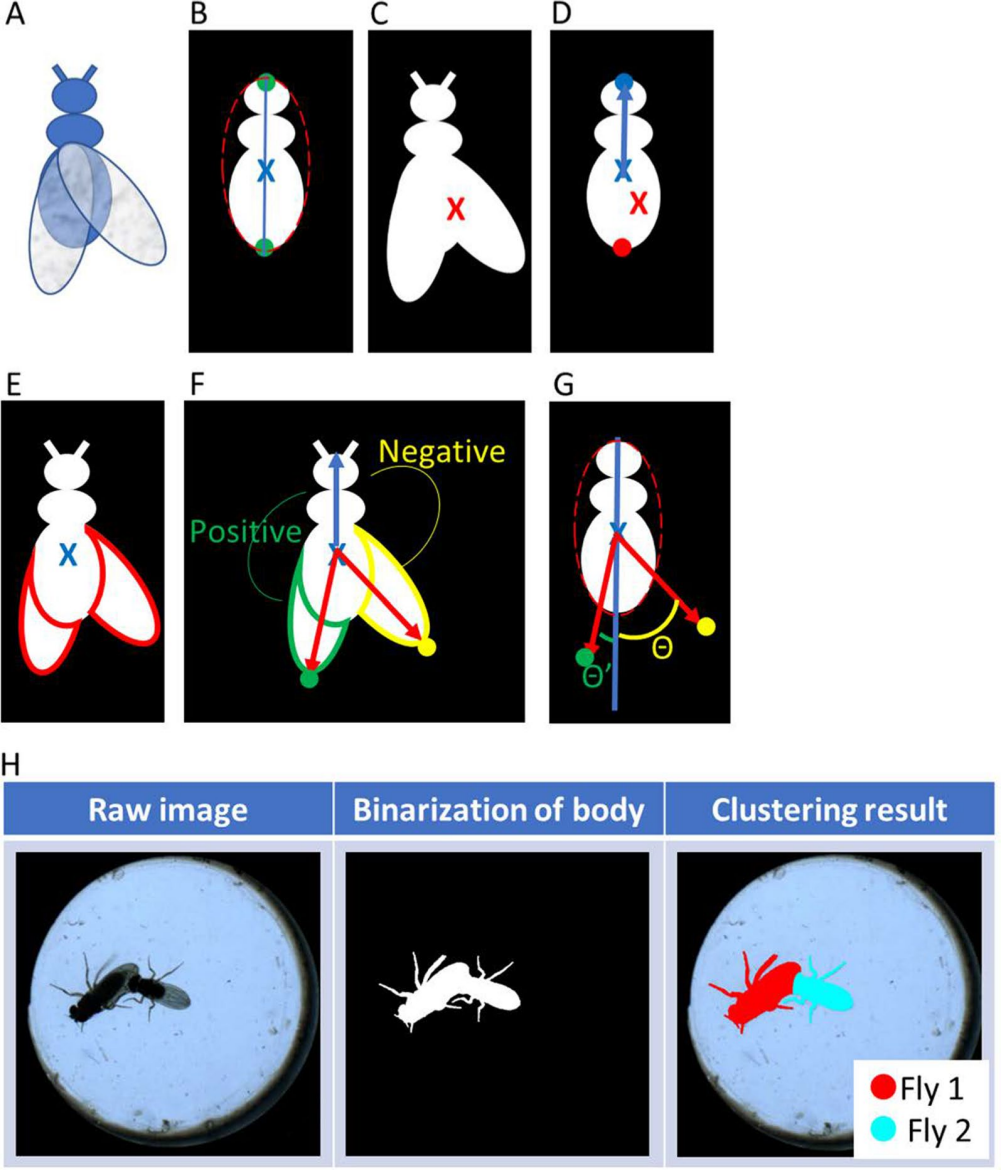
Wing/body characterization and spectral clustering. **A**–**D** Fly position and heading definition. **A** A schematic of a fly. **B** Torso ellipse fitting; the centroid of the torso (blue cross) denotes the position of the fly. The two ends of the major axis (green points) were assigned as the candidates for the head and tail. **C** The centroid of the whole body (red cross). **D** A candidate further to the centroid of the whole body is designated the head (blue point), and the other candidate is assigned to the tail (red point). The heading is the vector pointed from the centroid of the torso to the head point. **E**–**G** Wing extension angle calculation. **E** Edge of the wing calculation (red edge). **F** The wing is separated into right and left sides by a cross-product. The wing tips are defined as the farthest edge points from the centroid of the torso (the green and yellow points). **G** The wing extension angles (θ and θ’) between the wing tips and the major axis of the torso can thus be calculated. **H** The process of spectral clustering

After extraction of the torso and wings, the position and the heading of a fly were defined as shown in Fig. 3A–D. Figure 3A shows a fly model. The centroid of the torso was defined as the position of the fly (the blue cross in Fig. 3B). The major axis can be calculated by performing ellipse fitting to the region of the torso. The two ends of the major axis were defined as candidates for the head and tail (the green points in Fig. 3B). The red cross in Fig. 3C indicates the centroid position of the fly’s body. A candidate point farther from this red cross was defined as the head point (illustrated by the blue dot in Fig. 3D), while the other candidate point became the tail (depicted by the red dot in Fig. 3D). The heading of the fly can be obtained from the centroid of the torso to the head point (the blue vector in Fig. 3D).

### Wing/torso characterization - the wing extension angles (wing processing)

The wing extension angles were defined as shown in Fig. 3E–G. Edge detection was used to collect the pixels on the edge of the wing (red boundary in Fig. 3E). The wing vector was defined as pointing from the centroid of the torso to each edge pixel (red vector in Fig. 3F). The edge pixels can be divided into left and right sides by the cross product of the heading and wing vectors. The wing vector of the left edge pixels led to positive cross-product results with the heading vector (green boundary in Fig. 3F). Conversely, negative results are denoted on the right side (yellow boundary in Fig. 3F). After separating the edge points into two sides, the point farthest from the centroid of the torso on each side was defined as the wingtip (green point and yellow point in Fig. 3F). The wing extension angles (θ and θ’) between the wing tips and the major axis of the torso can thus be calculated (Fig. 3G).

### Identification and identity checkup

Three different identification modules were proposed to maintain high tracking accuracy in this system.

#### Rectilinear motion prediction

This study predicted the position of the fly by the rectilinear motion model according to the position of the fly in the previous two frames [4]. Tracking identity involved calculating the movement speed at known positions in two consecutive frames and using this speed to estimate where the fly might be in the third frame. Since the time between these three consecutive frames was very short (1/24 s × 2 = 1/12 s), the fly’s position closest to the predicted position was considered its true location. While both male and female flies had predicted positions, the closest fly to the predicted position was labeled the same identity.

#### Spectral clustering

When flies touched each other, the spectral clustering method was applied to differentiate the overlapping region according to the corresponding identity [27] (Fig. 3H).

#### Torso shape matching (head, thorax, and abdomen detection)

**Fig. 4:**
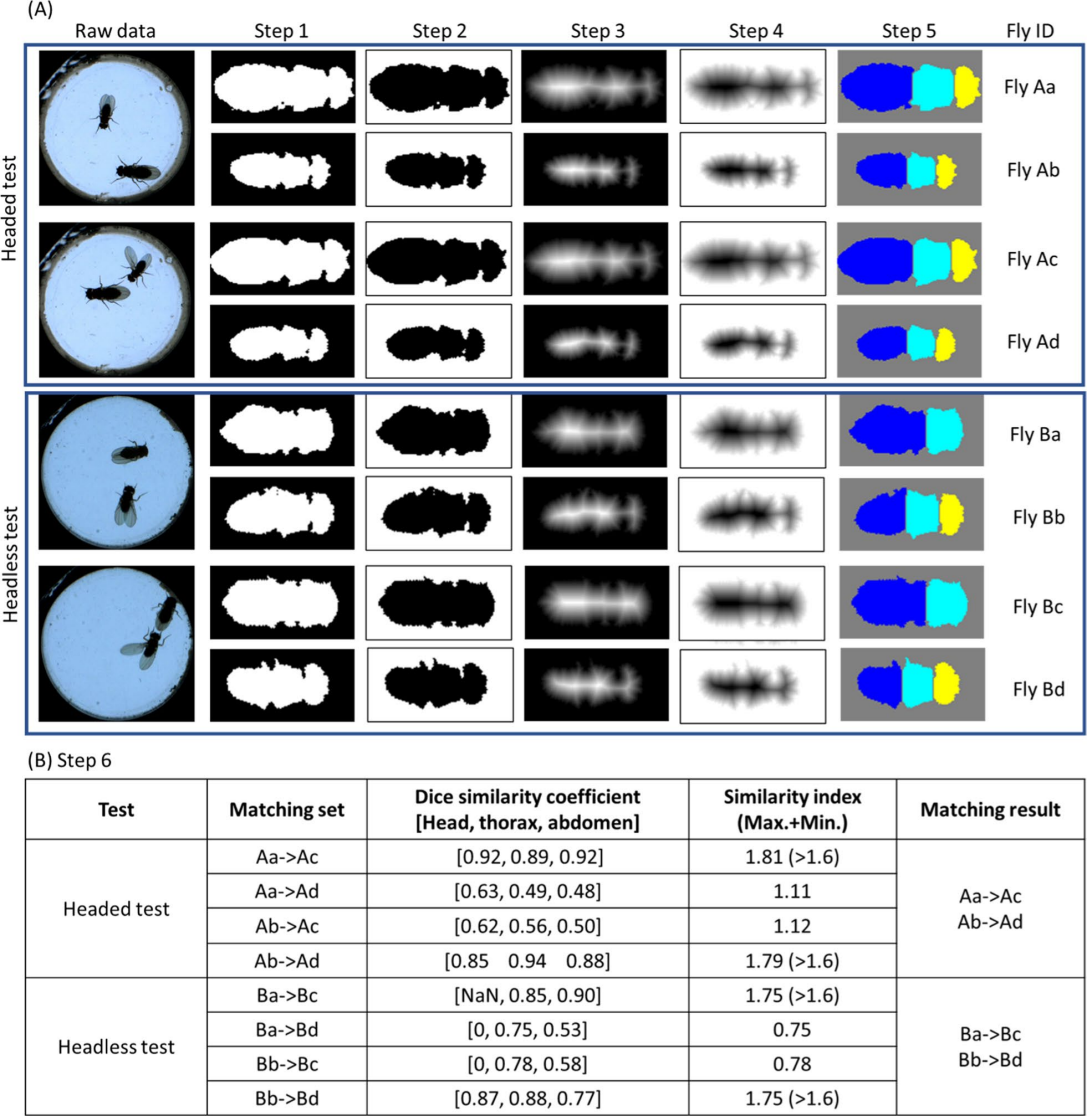
The steps of torso shape matching for head, thorax, and abdomen detection. **A** The process for a headed test and a headless test with two frames for each test is demonstrated. The different colors in step 5 denote the head, thorax, and abdomen. The female fly had a severed head in the headless test, while the female fly retained its head in the headed test. **B** The Dice similarity coefficient result of the comparison

To achieve greater accuracy when flies touch each other, the torso shape matching method was developed in this study (Fig. 4). The torso was segmented into head, thorax, and abdomen parts by watershed transform, and the identities were subsequently matched by the higher Dice similarity coefficient. This process (six steps) was executed every time the flies were separated after overlapping. Headed and headless tests are shown in Fig. 4A, with two frames in each test. The ‘headless test’ was used to monitor the courtship behavior of male flies toward females with severed heads. In comparison to normal females, females with severed heads exhibited no resistance to courtship by male flies, helping to minimize variables related to female rejection of male mating attempts. Figure 4B shows the matching results according to the Dice similarity coefficient.

- Step 1: Crop the binary image of the torso. Then rotate and align it horizontally.
- Step 2: Inverse the binary information.
- Step 3: Calculate the euclidean distance between each non-zero pixel and its nearest non-zero pixel.
- Step 4: Make an additive inverse of the result in Step 3. Thus far, the result was similar to an elevation map. Darker pixels indicated lower altitudes, such as valley regions. Relatively greater amounts of land can be considered to constitute watersheds that can separate nearby basins.
- Step 5: The head, thorax, and abdomen can be segmented based on the results of the watershed transformation.
- Step 6: The Dice similarity coefficient was computed between the corresponding part of the torso. Three similarity coefficients indicated the similarity of pairs on the head, thorax, and abdomen. A higher value denoted a more similar shape. The sum of the maximum and minimum of these three values must be greater than 1.6 to be considered the same identity.

Together, shape matching ensures the correct identity of flies when they overlap, and this approach can even be applied to headless flies without additional manual settings. It is important to emphasize that the torso matching method has certain limitations when dealing with abnormal fly postures during identity tracking. In instances where the fly was not in a normal standing position, such as when climbing a wall, torso matching may be unable to compute similarity indices. Although identity errors in such situations may not be immediately detected or corrected, flies do not continuously maintain abnormal postures, such as climbing on walls. Consequently, these errors in identity are identified and rectified during subsequent torso matching steps. Despite these limitations, the precision of identity tracking can be ensured through iterative identity verification processes. It is worth noting that the method proposed in this study can automatically detect the threshold utilized for watershed segmentation, alleviating the need for users to set additional parameters for torso segmentation.

### Behavior recognition

The image processing methods mentioned above can generate a correct position and enough feature descriptions of each fly. These parameters were used to identify each behavioral element, including singing, orientation, tapping, attempted copulation, and copulation during the courtship ritual.

#### Singing

Singing refers to the behavior in which the wing is spread open accompanied by vibrations, and the resulting wing sound resembles the behavior of singing for courtship. Due to the difficulty of audio recording, most studies monitored wing vibration to reflect singing. In accordance with previous reports [16, 28], a male with a wing extension angle greater than 30° was considered to be singing in this study.

#### Orientation

**Fig. 5:**
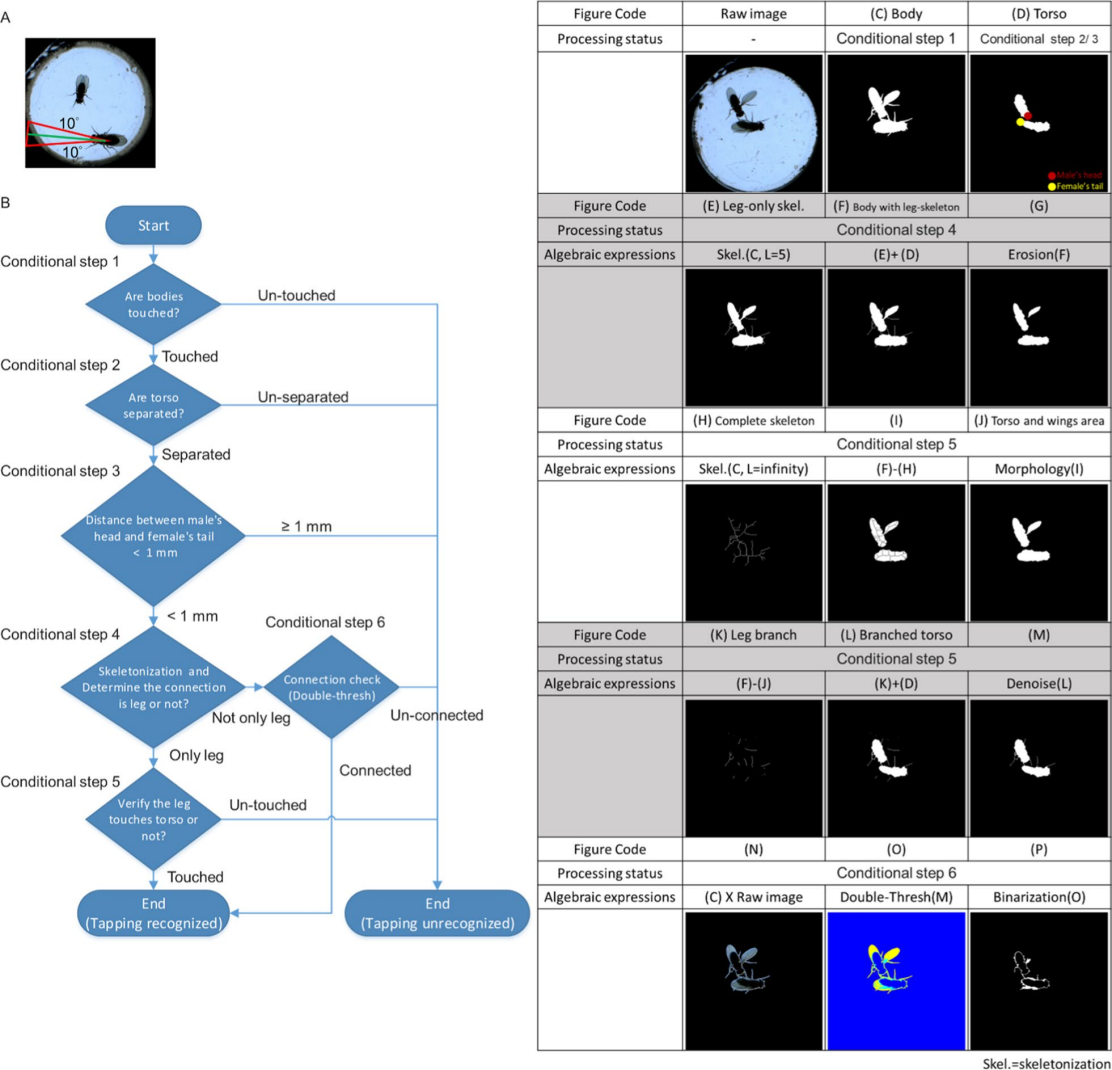
The solution to identify orientation and tapping behavior. **A** The definition of the orientation. **B** A flow chart of tapping detection. Six conditional steps are used to judge tapping behavior. **C**–**P** Demonstration of processing in each step. **C** Binarization of the body. **D** Binarization of the torso. **E** Leg-only skeletonization; the minimum branch length L of the skeletonization is set to 5 (L = 5). **F** Body with leg skeleton, the combination of (**E**) and the torso. **G** The erosion result of (**F**). **H** Completed skeleton, the skeletonization of the body (L = infinity). **I** The subtraction of the complete skeleton from leg-only skeletonization. **J** Torso and wings area, morphology processing result of (**I**). **K** Leg branch, the subtraction of the torso and wings area from the body with leg-skeleton. **L** Branched torso, the combination of the leg branch and torso. **M** Denoising result for a branched torso. **N** The multiplication of the binarization of the body and the raw image. **O** Double thresholding result of (**N**). **P** The binarizing and denoising results for the cyan area in (**O**)

The vector length from the centroid of the torso to the head was extended 2.5 times. Two expanding lines were determined by swinging the extending line ± 10°. A sector was defined as the field of view between these two expanding lines (red boundary in Fig. 5A). When the female was in this sector, the male was recognized as exhibiting orientation behavior.

#### Tapping

**Fig. 6:**
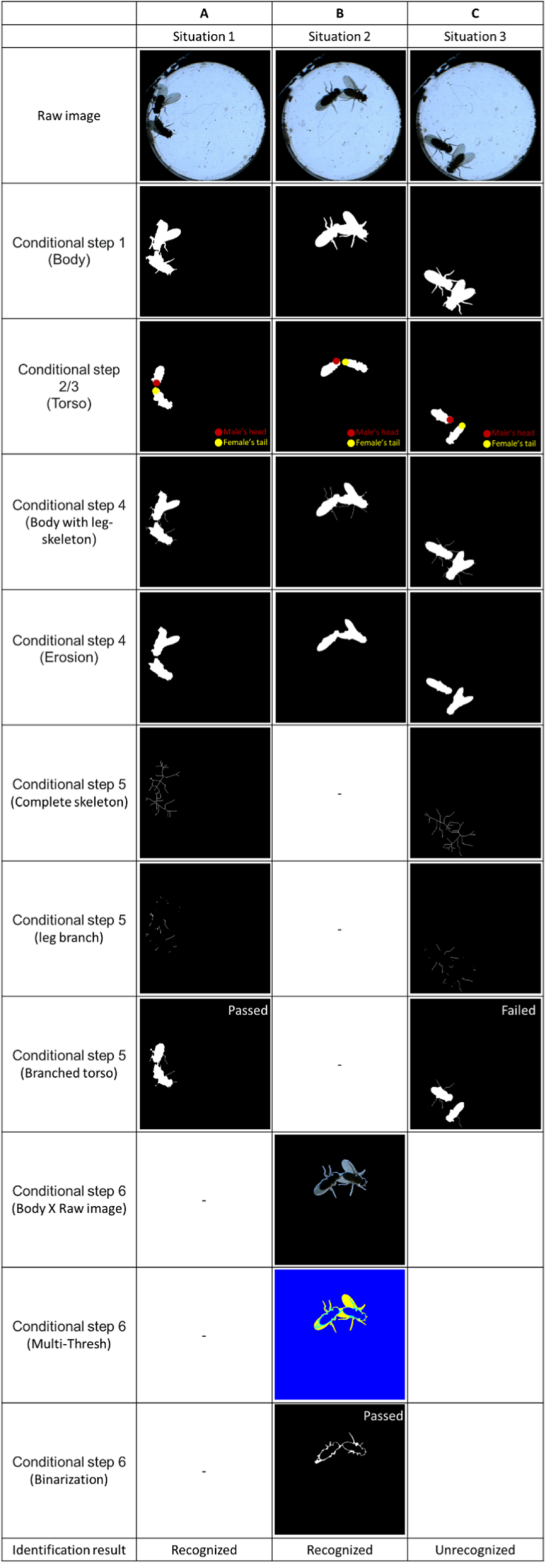
Demonstration of three tests with different connection situations. **A** Situation 1: Male touched female’s abdomen with his leg. Tapping behavior is recognized. **B** Situation 2: The male touched the female’s abdomen with his leg, but the event was under the wing of the female. Tapping behavior is recognized. **C** Situation 3: Male-touched female’s wing. Behavior is not recognized as tapping

Tapping behavior was defined as the time when a male touched a female’s abdomen with his leg. As shown in Fig. 5B, we gave a solution to determine the leg connection between two flies when tapping occurred. Figures 5C–P present the process at each step. Three tests are shown in Fig. 6 to display how different connection situations were identified via our method. Six conditional steps were applied to determine the leg connection (Fig. 5B).

- Conditional step 1: The body touching event must occur when tapping behavior occurs. The number of body regions was counted to capture touching events (Fig. 5C).
- Conditional step 2: The torso touching event does not occur when tapping occurs. The number of torso regions was counted to detect touching events (Fig. 5D).
- Conditional step 3: The tapping distance between the male’s head and the female’s tail (red and yellow points in Fig. 5D) was defined as less than 1 mm.

When the above criteria were met, there were only 3 connection situations. The tapping events were recognized in connection situations 1 and 2 but not in connection situation 3:

- Connection situation 1- Only the leg touched the female’s torso (Fig. 6A).
- Connection situation 2- The leg touched the female’s torso but under the wings (Fig. 6B).
- Connection situation 3- The leg touches the female’s wing but not the torso (Fig. 6C).
- Conditional step 4: Skeletonization was performed to reduce the object to branch. The minimum branch length L of the skeletonization was set to 5 to obtain the branch of the leg and maintain the shape of the wings and torso (Fig. 5E). Figure 5F shows the union of Fig. 5E and the torso (Fig. 5D). Subsequently, the skeletonized leg can be eliminated by using the erosion algorithm (Fig. 5G). While the connection disappeared after erosion, causing the region to separate, the connection part was considered the leg. The two possible connection situations involved the leg touching the torso or the leg touching the wing (Fig. 6A or C). On the other hand, while the region was still connected after erosion, two possible outcomes were wings covering the leg connections (Fig. 6B) or no touching (tapping) at all.
- Conditional step 5: This step was applied to determine if the leg touched the torso or wings (Fig. 6A or C). The minimum branch length L of the skeletonization was set to infinity to obtain the complete skeleton of the body (Fig. 5H). The entire skeletonization process simplified the fly’s body, primarily emphasizing its skeletal structure, akin to the process of focusing solely on the legs (Fig. 5E), while still retaining full leg details. Given that both processes successfully retain leg information, subtracting the complete skeleton from the leg-only skeletonization yielded a generalized outline of fly sans legs (Fig. 5I). After morphology processing, a more complete view of the shape of the torso and wings was obtained (Fig. 5J). The branch information of the leg (Fig. 5K) can be obtained by subtracting the torso and wing areas from the body with the leg skeleton. A branched torso can be generated by coupling the leg branch to the torso (Fig. 5L). After removing the noise (Fig. 5M), if the two branched torsos could be connected, the process was considered tapping (Fig. 6A). Otherwise, it is considered as no tapping behavior (Fig. 6C).
- Conditional step 6: This step was used to judge whether the wings were covering the leg (Fig. 6C) or if tapping was not occurring. Figure 5N is made by multiplying the binary image of the body by the original image. Otsu’s method was subsequently used to segment Fig. 5N with double thresholds to detect the connection of the legs (the cyan area in Fig. 5O). After binarizing and denoising the cyan area in Fig. 5O (Fig. 5P), if the two binarized regions are connected, tapping is considered to occur (Fig. 6B). If they were not connected, it was considered no tapping behavior. This method demonstrated robustness in obtaining leg information, with errors typically occurring only in scenarios where the original frame lacked complete leg information, such as when the fly’s legs were obstructed by the body or wings.

#### Attempted copulation

**Fig. 7:**
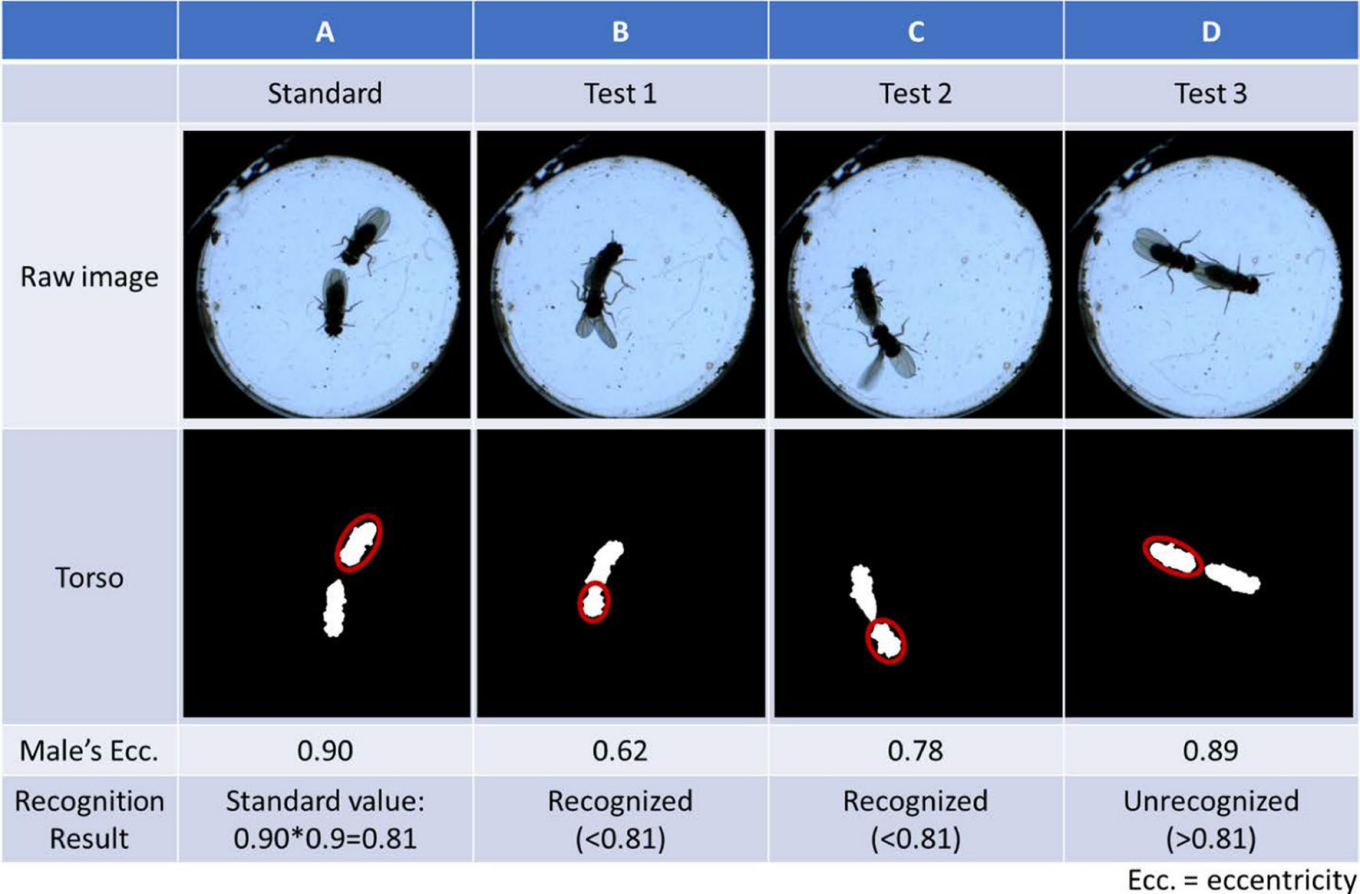
Attempted copulation was determined by the eccentricity of the male torso. **A** A frame in which the male does not bend his body is assigned as a standard figure. The standard value is defined as 0.9 times the eccentricity of a male’s torso. **B**, **C** The eccentricity of the torso is less than the standard value when a male bends his abdomen, whether the torsos are connected (**B**) or not connected (**C**). **D** If the male does not try to copulate with the female, the eccentricity of his torso will be greater than the standard value

In this study, ellipse fitting was performed on the torso of the male, and eccentricity was used to determine whether a male tried to mate with a female. Since males bend their abdomen and try to climb the female’s body during attempted copulation, the body of males changes from a long ellipse shape to a nearly round shape. Its torso eccentricity is thus much lower than the original shape. In each experiment, the user first selected a male without bending its body as a standard (Fig. 7A) and multiplied the eccentricity of the torso by 0.9 to obtain a standard value. In the following analysis, whether the torsos were connected or not, as long as the eccentricity was smaller than the standard value and the distance between the male’s head and the female’s tail was less than 1 mm, was regarded as attempted copulation (Fig. 7B and C). A number greater than the standard value was considered to indicate no attempted copulation (Fig. 7D).

#### Copulation

When the duration of attempted copulation was greater than half a minute, copulation behavior was determined. Since we did not study behavior after copulation in this report, the behavior of males in the rest of the test was considered copulation.

#### Noise filter

The rule of denoising is shown in the following:

1. All courtship behaviors were certified if the behavior was identified in more than 5 frames within 12 frames.
2. No behavior was determined when there was no behavior recognized for 12 continuous frames. Like in other courtship behaviors, the designation of “no behavior” requires a certain duration of performance to be defined as such. If any moment without defined behavioral occurrences is labeled as an instance of “no behavior”, the precision of this label would be compromised.
3. If one more behavior was recognized in the same frame, the priority of the recognition was copulation > attempted copulation > tapping > sing > orientation.

### Fly stocks and environmental details for the courtship assay

*D. simulans (D. sim.)* and *D. melanogaster (D. mel.)* Canton-S (CS) were obtained from the fly core in Taiwan. All flies were kept at 25 °C and 60% RH with a 12 h light/12 h dark cycle and were raised in standard white food supplemented with yeast, corn powder, agar, antibiotics, and preservatives. The flies were transferred to new vials with fresh food every 2–3 days until the behavioral assays were performed.

### Fly collection for courtship assay

Virgin CS females serving as courtship targets were collected within 8 h after emergence under CO_2_ anesthesia. Thirty female flies were housed in one vial for 7 days before the behavioral assays. For the dead females, the flies were decapitated immediately before the courtship test. Unless otherwise noted, all male flies were collected within 8 h after emergence under CO_2_ anesthesia. Thirty male flies were housed in one vial for 7 days before the courtship test. For flies of different ages, the flies were transferred to new vials every 2–3 days for 2, 8, 14, 21, 29, 35, 42, or 49 days before the courtship assay.

### Courtship assay

The diameter of the courtship arena was 11 mm. The wall of the arena and cover glass were coated with water repellent to prevent fly climbing. For courtship recording, 1 male and 1 female fly were placed into the arena under CO_2_ anesthesia. After a 5-min adaptation, the interaction between the males and females was recorded for 20 min. The recorded video was further analyzed to identify different male courtship behavioral elements.

*Total courtship time* The sum of all the behavioral elements, including orientation, tapping, singing, and attempted copulation. Data for males that had mated within 20 min were excluded.

*The proportion of behavioral elements* The time of each behavioral element divided by the total observation time. For analyzing males of different ages, the observation time was considered the time from the beginning to copulation for mated males or 20 min for unmated males. To compare mated and unmated males, considering that behaviors may change as a function of time, we analyzed mated and unmated males within a similar duration by analyzing mated males from the beginning of copulation and analyzing unmated males for 484.75 s, which is the mean duration for the copulation of mated males.

*The transition of behavioral elements* The number of changes from behavioral element A to B divided by the total number of changes from A to others. Again, since the transition probabilities may change as a function of time, we analyzed mated males from the beginning of the experiment to the time of copulation and analyzed unmated males for 484.75 s, the mean duration for the copulation of mated males.

### Statistics for courtship assay

All the statistical analyses were completed with Prism and MATLAB software. An unpaired Student’s t test was used to compare data between male courtship to live and dead females, between *D. mel* and *D. sim*, and between mated and unmated males. For the effect of age on courtship, regression analysis and quadratic fitting were performed via QR decomposition. For nonnormally distributed data, the Kruskal‒Wallis test followed by Dunn’s test was used to compare total courtship time, tapping, and the attempted copulation ratios. For normally distributed data, one-way ANOVA followed by Tukey’s test was used to compare the singing and orientation ratios.

## Results

### The system achieved high tracking accuracy and a high behavioral recognition rate

To evaluate our newly developed system, we first examined the tracking accuracy by comparing the system tracking results to the manually labeled data. An Intel core i7-9700F desktop processor (eight cores) processed at 3 GHz with 16 GB of random-access memory served as our evaluation platform. The 20 min of courtship test took just 35 min to complete the analysis. The average calculation speed was 0.07 s per frame. The MATLAB code is freely available for download at https://drive.google.com/drive/folders/1_3amhchUGGMqyJvftnIaTfoI34ArM6zE?usp=sharing.

Benefiting from the torso shape matching method, the identity tracking issue in a totally overlapped situation can be solved in this system. This study conducted detailed tracking on four different videos, randomly selecting 2500 instances for identity verification in each case. A total of 10,000 randomly chosen frames were analyzed, revealing only one instance of tracking error. This indicates a tracking accuracy of 99.99% for the system proposed in this study. The method introduced in this research performs identity verification based on the torso region, ensuring that errors in identity correspondence do not accumulate but are automatically corrected in subsequent verification steps.

**Fig. 8:**
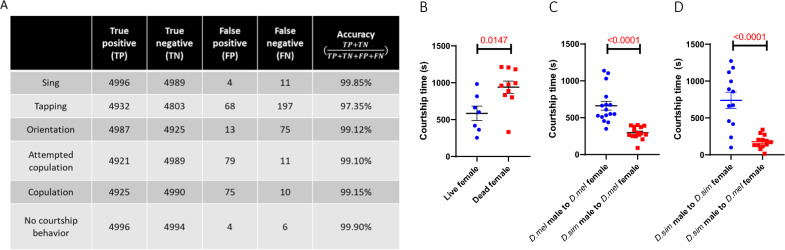
The system showed high accuracy and can be applied to detect different courtship behaviors. **A** Summary of the performance results obtained by comparing the recognition results to the manually labeled reference standard (5000 positive and 5000 negative data points for each behavioral element). **B** Total courtship time of males to live or dead females during the 20-min interaction. n = 7 for live females; n = 10 for dead females. **C** Total courtship time of *D. mel* or *D. sim* males to *D. mel* females during the 20-min interaction. n = 16 for *D. mel.*, n = 15 for *D. sim*. **D** Total courtship time of *D. sim* males to *D. sim* or *D. mel* females during the 20-min interaction. n = 12 for *D. sim*, n = 13 for *D. mel.* Student’s t test; mean ± SEM

We also examined the ability of the system to recognize behavioral elements by comparing the system recognition results to manually labeled reference data (5000 positive and 5000 negative data points for each behavioral element) (Fig. 8A). Among the six parameters, orientation, singing, attempted copulation, copulation and no courtship all had recognition rates greater than 99%. The rate of tapping was lower but still higher than 97.35%. A supplementary video (0.25 × speed) generated from our experimental trial shows the recognition of courtship behavior (Additional file 2). Together, the 99.99% identity tracking accuracy and greater than 97.35% behavioral element recognition rate guarantee the high reliability of this system.

To validate the applicability of the system, we then asked whether it could robustly detect courtship differences under different conditions. We first compared the courtship of CS males to live or dead (decapitated) females. To control all the tests under the same observation time, data for males mated within a 20-min interaction were excluded from the analysis of total courtship time. The system revealed that male flies performed more courtship to dead females than to live females (Fig. 8B). Since dead females were basically motionless, males had more opportunities to display courtship.

Next, we applied the system to compare courtship behaviors between different species, mainly *D. mel.*, and *D. sim.* As expected, because *D. mel.* females were used as the courtship target; only *D. mel.*, but not *D. sim.* males exhibited strong courtship behaviors (Fig. 8C). Since *D. sim* males also displayed a strong courtship tendency toward *D. sim* females (Fig. 8D), the decrease in courtship behaviors of *D. sim* males toward *D. mel* females was due to their lack of interest in other species rather than differences in courtship activities between the two species. In summary, these tests suggested that the system performed with high accuracy compared to the ground truth and can be applied to detect distinct courtship behaviors among different males or the same male to different females.

### Age-modulated courtship time and the proportion of behavioral elements

**Fig. 9:**
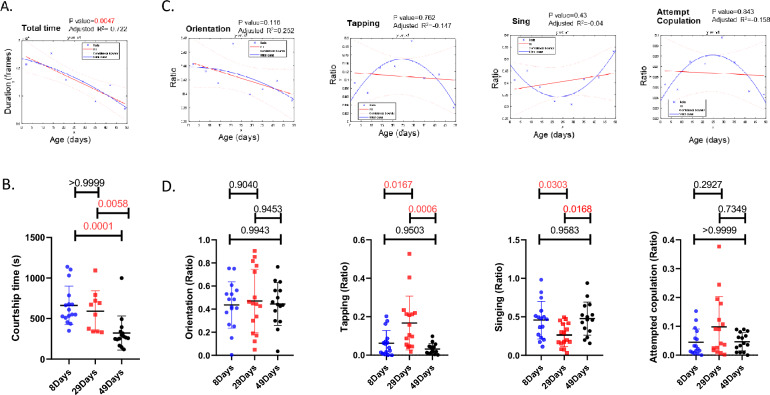
Courtship time and the proportions of behavioral elements changed with age. **A** The regression line (red) and quadratic curve (blue) for total courtship time over different ages. **B** The total courtship time of males aged 8, 29, and 49 days to females during the 20-min interaction. **C** The regression line (red) and quadratic curve (blue) for the ratio of orientation, tapping, singing, and attempted copulation over different ages. **D** The ratios of orientation, tapping, singing and attempted copulation of males at 8, 29, and 49 days old to females. n = 13 for 2 days old, n = 16 for 8 days old, n = 11 for 14 days old, n = 16 for 21 days old, n = 10 for 29 days old, n = 13 for 35 days old, n = 15 for 42 days old, n = 15 for 49 days old. QR decomposition was applied for regression analysis and quadratic fitting. One-way ANOVA followed by Tukey’s test was used to compare the signaling ratio and orientation ratio among flies at 8, 29, and 49 days of age, and the Kruskal‒Wallis test followed by Dunn’s test was used to compare courtship time, tapping ratio and attempt copulation ratio among flies at 8, 29 and 49 days of age. Mean ± SEM

This newly developed system allowed us to examine a large number of fly courtship events under different conditions. For example, aging is known to affect animal behavior and physiology [2]. As expected, by examining the courtship of males of different ages to 8-day-old females, we observed a significant decrease in the total courtship time based on linear regression (Fig. 9A). Compared to young and middle-aged males, 49-day-old males exhibited very little courtship behavior (Fig. 9B). We also examined the proportion of each behavioral element, which was calculated by normalizing the duration of each behavioral element to the total observation time. Surprisingly, despite the decrease in courtship time, there was no significant linear trend in the proportions of the behavioral elements (Fig. 9C). Instead, we noticed that there were concave curves for tapping and attempted copulation, as well as a convex curve for singing in terms of the relationship between age and behavioral proportion, suggesting that middle-aged flies mainly displayed tapping and attempted copulation rather than singing. Correspondingly, 29-day-old males performed significantly more tapping and attempted copulation but significantly less singing than did 8-day-old and 49-day-old males (Fig. 9D). Taken together, these data showed that age can significantly modulate fly courtship behaviors. Interestingly, while total courtship decreased with age, concave/convex patterns of behavioral elements suggested more tapping and attempted copulation but less singing in middle-aged flies than in young and old flies.

### Males that successfully mate exhibit courtship patterns different from those of males that fail to copulate

**Fig. 10:**
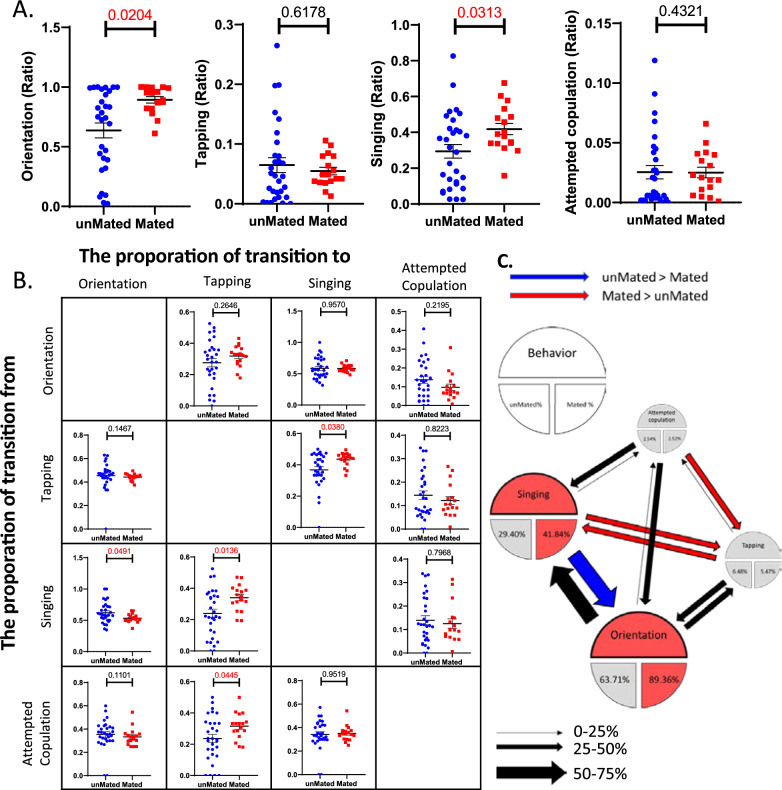
Males with or without successful mating showed distinct courtship proportions and behavioral sequences. **A** The ratios of orientation, tapping, singing, and attempted copulation of males with or without successful mating. n = 30 for unmated, n = 17 for mated; **B** Behavioral transition matrix for mated and unmated males interacting with females. **C** The courtship patterns for mated and unmated males interacting with females. The sizes of the pie area and arrow reflect the ratio of behavioral elements and the proportion of transitions, respectively. For each pie chart, the mean ratio of each behavioral element is shown for mated (right) and unmated (left) males. Red denotes significantly greater performance in mated males. Blue denotes significantly greater performance in unmated males. n = 30 for unmated, n = 17 for mated; Student’s t test; mean ± SEM

Finally, we were interested in knowing whether there was a difference in courtship behaviors between males with or without successful mating during 20-min interactions. We thus compared the proportion and transition of courtship elements between successfully mated males and unmated males. For the proportion of courtship elements, mated males showed more orientation and singing than unmated males (Fig. 10A). For the transition matrix, which calculates the proportion of change among different behavioral elements, mated males underwent a greater switch from attempted copulation to tapping and more transitions between singing and tapping, while unmated males underwent a greater switch from singing to orientation (Fig. 10B). The courtship patterns based on the proportions and transitions of these behavioral elements are illustrated in Fig. 10C.

## Discussion

In this study, we applied an image processing technique to establish a new system to detect and analyze fly courtship automatically. Three important features were developed in our system. First, in contrast to machine learning methods, our system based on image processing requires no model training and can be easily adopted. Second, by using the torso part to assign the identity, a robust solution was given to minimize the overlap issue and maintain high identity tracking accuracy (99.99%). Third, new methods were introduced to detect tapping and attempted copulation, which resulted in a high recognition rate of detailed courtship elements (> 97.35%). The output results were strongly correlated with ground truth data and can be applied to detect distinct courtship behaviors toward different female targets or courtship performed by different males. The analyzed data included total courtship time, the proportion and transition of courtship elements, which together presented a complex and dynamic behavioral pattern. With appropriate cameras and lenses, this method is scalable, allowing for tracking in arenas of various sizes without constraints. It also enables simultaneous tracking in multiple arenas. This versatility makes it suitable for fly research projects in laboratory settings and applicable in school courses. It can serve as a research project topic or be integrated into course curricula, accommodating students with varying levels of experience.

Machine learning methods have been applied to analyze flies’ social behaviors in previous studies. The advantage of machine learning is that it does not require behavioral definition. For example, Ross Mckinney employed the Scikit-learn AdaBoost decision tree classifier for the detection of mating behavior in flies [21]. Pereira et al. utilized more than 30 state-of-the-art neural network backbones and modular network architectures (deep learning systems) for multianimal pose tracking [17]. JAABA employs a boosting algorithm to train classifiers for multiple behaviors [12]. Klibaite et al. adopted unsupervised machine learning methods to quantify the behavior of paired flies [14]. However, the performance of systems based on machine learning heavily relies on the quality of the hardware. The process also requires the accumulation of a substantial amount of manually labeled files and the confirmation of model convergence before conducting pre-experimental preparations. Some systems even need to introduce behavioral definitions again to increase accuracy [15]. Moreover, when the machine learning system is used under different setups with a new environment, the previously trained model is not applicable and requires more training procedures to establish a new model, which means additional effort to recollect and relabel the training data manually. In contrast, methods based on image processing require no model training and require only minor adjustments of the values for behavioral definitions under new setup conditions, providing an easier and more accessible approach to analyzing animal behaviors, such as fly courtship elements.

There have been several groups developing systems for fly tracking based on imaging process, including IowaFLI Tracker [10], Ctrax [1], and others [20, 23, 26]. Unfortunately, most of these studies placed greater emphasis on the extraction of locomotion-related behaviors, such as fly position and head direction. Straightforward locomotion-associated behavior determination cannot be further applied to complex behavioral assessments, such as courtship behavior. However, our study utilized image processing techniques as its foundation, achieving behavior monitoring beyond the confines of locomotion types and yielding exceptionally high recognition rates for complex courtship behavior.

Notably, in the field of fly behavior detection, the issue of misidentification due to overlapping has seldom been addressed. Since the appearances of flies are similar, overlapping is always an issue for ensuring the accuracy of tracking identification. Previous studies often fitted a two-component Gaussian mixture model to pixel location and brightness to handle abutting flies [6, 15] or applied the integrated K-means method to separate slightly overlapped regions [3]. However, heavily overlapping situations, such as a fly climbing on top of each other, still cannot be resolved clearly. In this study, we assigned fly identity by extracting and matching the head, thorax, and abdomen parts to the reference. This torso matching method allowed us to track flies with a high accuracy rate after overlapping situations.

Tapping is an important courtship element for male to evaluate female condition based on cuticular pheromones [7]. This behavior, however, is very subtle and cannot be easily detected by previously reported systems. By examining leg connections to the torso via image processing, we can successfully recognize this delicate behavior automatically. In addition to tapping, we also quantified the eccentricity of torso bending to determine attempted copulation. The definition of this behavioral element is now more precise and physically meaningful than that used in previous studies.

Aging has a significant impact on fly behaviors, including courtship activity [2]. Our data also confirmed that total courtship decreases with age. However, by examining each behavioral element in detail, we surprisingly found that the ratio of each element did not change linearly with age. Instead, some behaviors are more common in middle age than others. These findings imply that as age increases, male flies may need to adjust their courtship strategy in response to changes in their physical condition. Intriguingly, after middle age, they altered their behaviors again to fit their physiological deterioration. A peak performance in late adulthood is uncommon but not unique. Several phenotypes like this have been reported, including some cognitive functions in humans [9] and some functional tasks in mice [24]. How flies adjust their decisions based on physiological changes with age will be interesting for further investigation.

We also compared courtship behaviors between mated and unmated males, the pattern of which can be illustrated based on the proportion and transition matrix of courtship elements (Fig. 10C). For both mated and unmated males, many exchanges occurred between orientation and singing, and these two behaviors constitute most of the courtship. There was also some exchange between singing and tapping. The probabilities of three behaviors (orientation, singing, and tapping) leading to attempted copulation were relatively similar. The differences between mated and unmated males are also illustrated in this scheme. Compared to unmated males, mated males spent more time on orientation and singing. After singing, mated males were more likely to perform tapping, while unmated males were more likely to orientation. Overall, we observed more dynamic exchanges among behavioral elements in mated males than in unmated males. While these distinct courtship patterns may reflect different female responses, they may also contribute to the different results of mating success.

Although our program can successfully identify major behavioral elements, there is still potential room for further improvement. For example, licking behavior is usually considered part of courtship [7] but cannot be detected clearly in our current setting. Detection of such delicate behavior requires a much higher magnification of the lens. In addition, female behaviors were not considered in this study. Although courtship is a male behavior, female responses play an important role in affecting male decisions. Therefore, the distinct courtship patterns between mated and unmated males in our analysis may be due not only to differences in male strategies but also to differences in female responses. The use of immobilizing or dead females is one way to exclude female influences. However, to reveal real interactions between two sexes, a live female is an indispensable target for male courtship. Females exhibit various behaviors, including several rejection behaviors, in response to male courtship [8]. Automatic recognition of these behaviors will be much more difficult than male courtship and will be the main challenge for subsequent system development.

## Conclusions

Fruit fly courtship behaviors have always been an important model for behavioral research in the laboratory. While previous research has focused mostly on total courtship time, our studies demonstrated that males also display distinct courtship patterns, which information requires a detailed analysis of behavioral proportions and transitions. Our new system therefore played a major role here in allowing us to investigate each behavioral element thoroughly. We believe that further investigations of these courtship elements as well as courtship patterns would provide us with additional information about this social behavior, from the molecular or neural mechanism to the functional significance. Our automated system, or more advanced program, will be of crucial importance for these studies in the future.

### Supplementary Information

**Additional file 1.**Supplementary Information.

**Additional file 2.**Supplementary Video.

## Data Availability

The data supporting the findings of this study are available within the paper and its Supplementary Information and upon request from the corresponding authors.
