# Supplementary material for: An automatic system for recognizing fly courtship patterns via an image processing method
Source: Behav Brain Funct. 2024 Mar 16;20:5. doi: 10.1186/s12993-024-00231-4 (PMC10943763; doi:10.1186/s12993-024-00231-4)
Supplement: Supplementary file 1 — Additional file 1.Supplementary Information. [file 12993_2024_231_MOESM1_ESM.pdf]

Supplementary Information: Experimental parameter setting guidelines

1. Setting of Experimental Parameters:

The primary objective of this study was to propose a method for accurately tracking identity and recognizing mating behavior. The experimental settings are adjustable based on user preferences, and the following guidelines are provided for optimal configuration.

(1) Light Field Environment Contrast

The suggested light field setting in this system is backlit, without specific requirements for intricate details on the fly's body. Consequently, the contrast between light and shadow is adjusted to such a level that the human eye can effectively distinguish between the background and the fly's body and legs.

(2) Video Format for Loading

Given the diverse range of video decoding formats, the system presented in this study processes images. Users can record videos using their cameras, manually split all frames, input the frames per second (FPS) value into the system, and subsequently load all frames into the system.

(3) FPS Settings during Experimental Recording

For optimal results, this study recommends an FPS setting of 24 or higher. The FPS camera setting used in this study was also 24. The accuracy of identity tracking is directly influenced by FPS. In cases of excessively low FPS, the continuity of fly movement and behavior becomes challenging to determine reliably. Considering the maximum moving speed of a male fly (18.24 mm/s) and the length of the fly (approximately 3.5 mm), if the movement distance between each frame is less than 1/3 of its body length, an FPS setting of 15.6 or above is advised. Users can adjust the FPS based on their specific requirements following this principle.

(4) Fly Size Parameter Requirements (e.g., Lengths and Width)

This study employed flies with a length of 3.5 mm or above and a width of 1 mm. If users prefer smaller flies, they can achieve this by selecting a larger magnification lens. Clear imaging of the fly's legs is essential for effective analysis.

(5) The Angle Definition for "Singing" Behavior

Users have the flexibility to adjust these values in the program settings based on their specific circumstances. The definition of "singing" behavior, specifically the requirement of a 30-degree wing spread, is derived from the definition provided by (von

Philipsborn et al., 2011; O'Sullivan et al., 2018).

2. Graphical User Interface Setting Guidelines:
Supplementary Figure 1 depicts the GUI interface during program execution. For the wing area, the intensity threshold was set at 80, the similarity index was set at 1.6, and the eccentricity of the torso was multiplied by 0.9. These values are based on empirical data, and users can directly modify them according to their preferences through the GUI interface.

(1) Wing Extraction Coefficient
After obtaining the complete representation of the fruit fly body, the RGB color space of the fly body was divided by 80 and subsequently multiplied. After rounding, this process generally allows straightforward separation of the wing and torso areas. As the RGB color space values for the wings are significantly greater than those for the torso, users can adjust these values based on their specific imaging conditions.

(2) Similarity Coefficient
A similarity index of 1.6 was used to identify the fruit fly based on the corresponding values for the head, thorax, and abdomen of the torso. A value closer to 2 indicates a closer correspondence of the torso. When the suggested baseline for this value is 1.6, users can customize it according to their specific requirements.

(3) Torso Eccentricity Threshold
The eccentricity of the torso multiplied by 0.9 was used to determine whether a male fruit fly bent its body. When a male fruit fly bends its body, the shape of the torso deviates from an elliptical form, resulting in a decreased eccentricity. This metric is used to ascertain whether a male fruit fly has arched its torso, and users can set this threshold according to their specific preferences.

Users can easily load video data from a designated folder, set the threshold for background subtraction, and define the threshold for noise removal (areas smaller than the specified threshold are considered noise and are removed). Camera-related parameters such as the FPS during recording, the ratio between the image and the actual scale (number of pixels per mm), the similarity index, and the wing extraction coefficient can be configured. Finally, users can define mating behavior and specify the output folder to initiate the analysis.

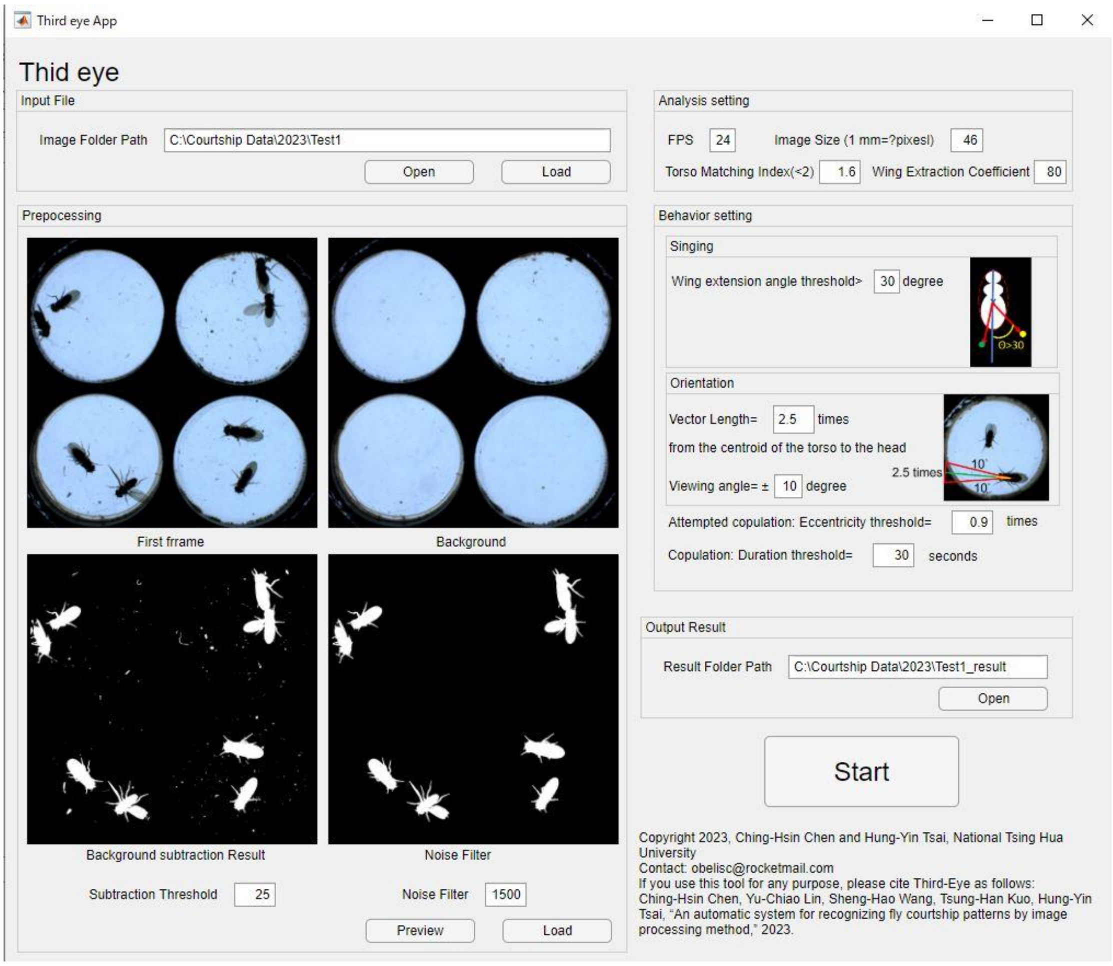

Supplementary Figure 1: The GUI of this system

Users can adjust the threshold values for background subtraction, noise reduction parameters, and settings based on the actual FPS and image size. The parameters defining courtship behavior can also be configured according to user requirements.

Reference:

O'Sullivan, A., Lindsay, T., Prudnikova, A., Erdi, B., Dickinson, M., and von Philipsborn, A. C. (2018). Multifunctional Wing Motor Control of Song and Flight. *Curr. Biol.* 28. doi: 10.1016/j.cub.2018.06.038.

von Philipsborn, A. C., Liu, T., Yu, J. Y., Masser, C., Bidaye, S. S., and Dickson, B. J. (2011). Neuronal Control of *Drosophila* Courtship Song. *Neuron* 69. doi: 10.1016/j.neuron.2011.01.011.
